# Engineering a palette of eukaryotic chromoproteins for bacterial synthetic biology

**DOI:** 10.1186/s13036-018-0100-0

**Published:** 2018-05-10

**Authors:** Josefine Liljeruhm, Saskia K. Funk, Sandra Tietscher, Anders D. Edlund, Sabri Jamal, Pikkei Wistrand-Yuen, Karl Dyrhage, Arvid Gynnå, Katarina Ivermark, Jessica Lövgren, Viktor Törnblom, Anders Virtanen, Erik R. Lundin, Erik Wistrand-Yuen, Anthony C. Forster

**Affiliations:** 1 Department of Cell and Molecular Biology, Uppsala University, Uppsala, Sweden; 2 iGEM Uppsala, Uppsala University, Uppsala, Sweden; 3 Biology Education Centre at Uppsala University, Uppsala, Sweden; 4 Department of Medical Biochemistry and Microbiology, Uppsala University, Uppsala, Sweden; 5 Science for Life Laboratory, Uppsala University, Uppsala, Sweden

**Keywords:** Chromoprotein, Fluorescent protein, Coral, *Escherichia coli*, Genetic marker, Reporter gene, Integration, Fitness cost, BioBrick, iGEM

## Abstract

**Background:**

Coral reefs are colored by eukaryotic chromoproteins (CPs) that are homologous to green fluorescent protein. CPs differ from fluorescent proteins (FPs) by intensely absorbing visible light to give strong colors in ambient light. This endows CPs with certain advantages over FPs, such as instrument-free detection uncomplicated by ultra-violet light damage or background fluorescence, efficient Förster resonance energy transfer (FRET) quenching, and photoacoustic imaging. Thus, CPs have found utility as genetic markers and in teaching, and are attractive for potential cell biosensor applications in the field. Most near-term applications of CPs require expression in a different domain of life: bacteria. However, it is unclear which of the eukaryotic CP genes might be suitable and how best to assay them.

**Results:**

Here, taking advantage of codon optimization programs in 12 cases, we engineered 14 CP sequences (meffRed, eforRed, asPink, spisPink, scOrange, fwYellow, amilGFP, amajLime, cjBlue, meffBlue, aeBlue, amilCP, tsPurple and gfasPurple) into a palette of *Escherichia coli* BioBrick plasmids. BioBricks comply with synthetic biology’s most widely used, simplified, cloning standard. Differences in color intensities, maturation times and fitness costs of expression were compared under the same conditions, and visible readout of gene expression was quantitated. A surprisingly large variation in cellular fitness costs was found, resulting in loss of color in some overnight liquid cultures of certain high-copy-plasmid-borne CPs, and cautioning the use of multiple CPs as markers in competition assays. We solved these two problems by integrating pairs of these genes into the chromosome and by engineering versions of the same CP with very different colors.

**Conclusion:**

Availability of 14 engineered CP genes compared in *E. coli*, together with chromosomal mutants suitable for competition assays, should simplify and expand CP study and applications. There was no single plasmid-borne CP that combined all of the most desirable features of intense color, fast maturation and low fitness cost, so this study should help direct future engineering efforts.

**Electronic supplementary material:**

The online version of this article (10.1186/s13036-018-0100-0) contains supplementary material, which is available to authorized users.

## Background

Coral reefs are colored by fluorescent proteins (FPs) and chromoproteins (CPs) that constitute a homologous eukaryotic protein family with the jellyfish green FP (GFP) [1, 2]. These GFP homologs are small proteins each encoded by a single gene, comprise a relatively high percentage of soluble proteins in expressed tissues, and form their chromophore without needing cofactors or substrates other than oxygen. Such properties facilitated their cloning and engineering to revolutionize imaging in vivo. In contrast to FPs, CPs absorb visible light intensely to give colors clearly visible under ambient light and almost all have low fluorescence [1, 2].

CP absorption properties endow CPs with certain advantages over FPs, such as instrument-free detection by eye, efficient FRET quenching and photoacoustic imaging [3, 4]. Detection of FPs requires an ultra-violet light (UV) lamp, fluorometer or flow cytometer and can be limited by background fluorescence, photobleaching and UV damage of the sample. An alternative popular genetic reporter, the lux gene cluster, requires a luminometer for detection. CP detection is also advantageous over traditional colorimetric assays such as lacZ, which require expensive exogenously-added substrate and can be limited by background from endogenous enzyme [5]. CPs are thus particularly attractive as markers in living organisms [6–8], for the annual international Genetically Engineered Machine (iGEM) competition and teaching [9], as dye replacements, for art, and for cell biosensor applications in the field where costs and low resources are important considerations. Current methods for detecting environmental, agricultural and food contaminants, landmines and biowarfare agents, and medically-relevant targets can be improved by synthetic biology [10]. For example, bacteriophage have been engineered to cause bioluminescence of pathogenic bacteria in food, and bacteria have been engineered to fluoresce upon detection of spoiled meat gas [11], trinitrotoluene (TNT) products [12] or arsenic [13]. Adaptation of these biosensors to non-fluorescent detection for use in supermarkets or the field beckons, but it is unclear which CP genes might be suitable or how best to assay them quantitatively.

While the GFP family is native to eukaryotes, most foreseen near-term applications of CPs require efficient expression in bacteria such as *E. coli* where engineering is more straightforward. Such efficient heterologous expression often requires codon optimization, a mostly proprietary process that is still more of an art than a science, necessitating validation in each case [14]. Some CP genes are available commercially, but items have been discontinued without warning (e.g. fwYellow) and they lack the characterization and free availability associated with publication. CPs from ATUM cost $225/gene [15] and contain unwanted (“illegal”) restriction sites that interfere with the popular, standardized, BioBrick cloning method [16], while our CPs made available via the Registry of Biological Parts [16] incur their $500 annual fee. Furthermore, just as FP comparisons were needed to determine which FPs were best for engineering and certain applications [17], CPs need to be compared and their properties and assays improved.

CP publications to date have not reported on bacterial cell toxicity and typically focus on an individual CP ([2, 15, 18–22] in Table 1), making a survey of the relative properties of CPs and their genes difficult. Of the 11 non-synthetic CP genes listed in the right seven columns of Table 1, all were expressed solely from their native eukaryotic DNA sequences, with only four reported to be expressed and matured highly enough in *E. coli* to give intensely-colored colonies (asPink, amilGFP, aeBlue and amilCP). Thus we considered that, for some of the other seven native CP genes, codon optimization of the eukaryotic sequences to match *E. coli* preferences might be necessary for high functional expression in bacteria. Here, to simplify and expand CP study and applications, we make available 14 engineered CP genes that are functionally expressed, characterized and compared in *E. coli*, together with chromosomal mutants suitable for competition assays.

**Table 1:**
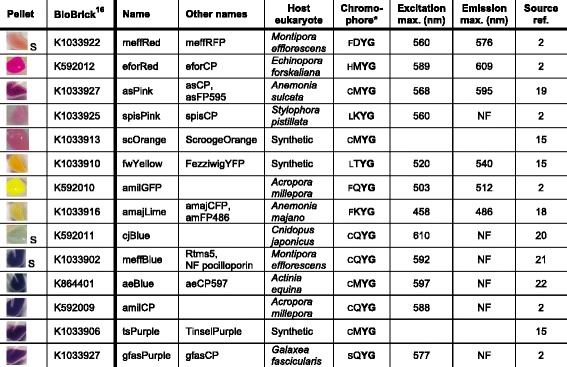
Bacterial pellets and spectroscopic characteristics of the 14 CPs in this study. The right seven columns give the CP sources and reported spectroscopic properties; only three of these genes had been codon optimized (Synthetic in fifth column). The left two columns show *E. coli* pellets expressing our BioBrick plasmid clones beside their ID numbers; only the amilGFP and amilCP plasmids were not codon optimized (see Fig. 1a and b)

* Smaller font denotes the amino acid at position 64 (GFP numbering; one of two positions we selected for mutation in amilGFP and amilCP) immediately upstream of the chromophore tripeptide sequence

*NF* Non-fluorescent

S Slower color development

## Results and discussion

### Recoding of CPs for expression as markers in *E. coli*

**Fig. 1:**
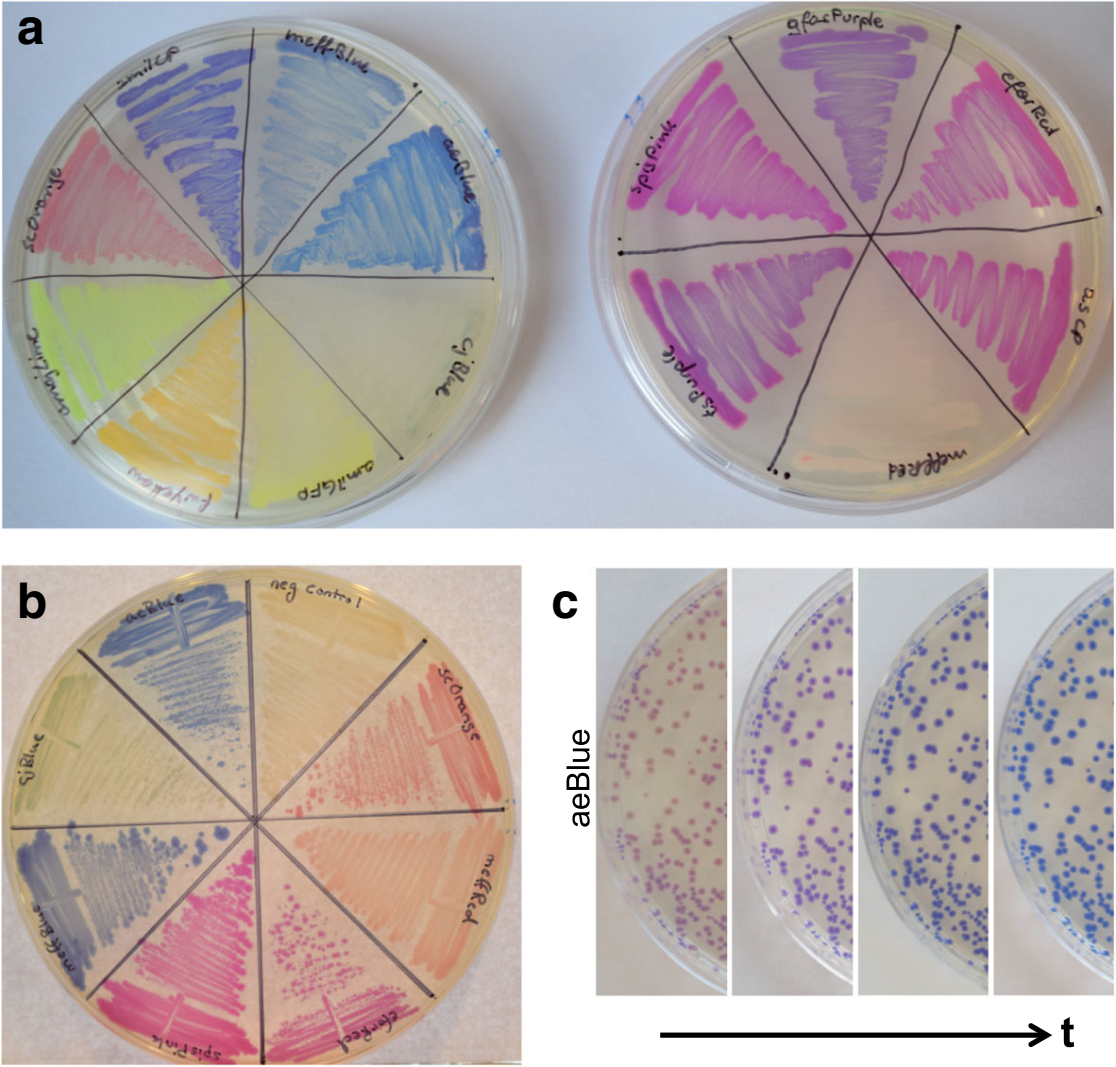
Comparison of CP color development. **a** LB chloramphenicol agar plates incubated at 37 °C for 20 h comparing (left, clockwise starting from 12 o’clock) meffBlue, aeBlue, cjBlue, amilGFP, fwYellow, amajLime, scOrange, amilCP and (right, clockwise starting from top) gfasPurple, eforRed, asPink (asCP), meffRed, tsPurple and spisPink expressed from a high-copy plasmid. **b** Comparison of cjBlue and meffRed with similarly-colored CPs after full color development = 37 °C for four days (clockwise starting from 12 o’clock: control promoter-less aeBlue that also lacked a ribosome binding site, scOrange, meffRed, eforRed, spisPink, meffBlue, cjBlue and aeBlue). **c** Time dependence of the color of aeBlue. A single plate is shown after: 19 h at 37 °C, then additional 4 °C incubations for one, two and three days (left to right, respectively)

All CP genes (Table 1), except the native amilGFP and amilCP genes, were codon optimized for plasmid-based expression in *E. coli* by proprietary computational programs and synthesized commercially without illegal BioBrick restriction sites (to conform with BioBrick standard RFC10, the most common gene assembly standard in synthetic biology). The genes encoding amilGFP and amilCP were amplified by the polymerase chain reaction (PCR) from the plasmids pGEM-T-11 and pGEM-T-14 [6] and illegal BioBrick sites were removed by mutagenesis. The resulting 14 BioBrick genes were ligated to a medium constitutive promoter in medium- and high-copy plasmids (see METHODS). After transformation into *E. coli*, the bacterial colonies on agar plates had strongly visible colors under ambient lighting. However, meffRed, cjBlue and, to a lesser extent, meffBlue required substantially longer incubation due to slower color development (Fig. 1a and b). Liquid cultures of all 14 CP strains yielded strikingly-colored pellets when centrifuged (Table 1, left column). More aeration tended to give more intense colors, consistent with knowledge that chromophore maturation in all GFP-like proteins is dependent on reaction with oxygen [1]. Maturation times measured by anaerobic growth overnight then opening the flasks to air were comparable with those of FPs: a commonly-used red FP (mRFP1), aeBlue and amilCP gave t_1/2_ ~ 22, 24 and 54 mins, respectively (but meffRed and cjBlue were much slower; see METHODS). Interestingly, one CP, aeBlue, changed color over time (Fig. 1c), indicating multi-step maturation. We concluded that recoding for expression in bacteria was successful in producing colored bacteria in every case and that these CP genes can be used as visual markers for plasmid transformation and cloning in *E. coli* (e.g. Additional file 1: Figure S2A discussed below).

### CP genes as quantitative reporters of gene expression

Quantitation of FPs has been better than for CPs due to a longer development time and compatibility with sophisticated fluorescence instruments including flow cytometers. However, there is a niche for CPs as reporters that could be semi-quantitated readily by eye without equipment (see BACKGROUND), and the potential for more accurate quantitation of cellular expression using cameras or spectrophotometers together with appropriate software.

**Fig. 2:**
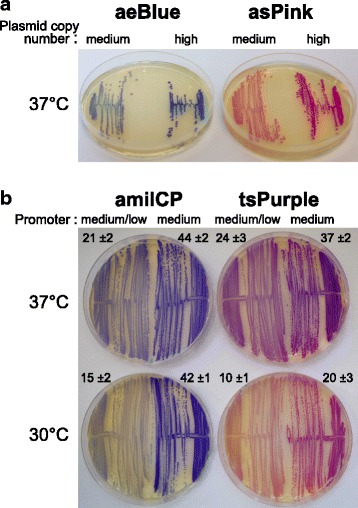
Variation in color intensity with CP expression. **a** Effect of plasmid copy number (using J23110 promoter). Medium copy plasmid indicates pSB3K3; high copy, pSB1K3. Plates were incubated for 20 h. **b** Effect of promoter strength and temperature (using high copy plasmid). Left side of plates: medium/low promoter BBa_J23116 (396 arbitrary units [16]). Right side of plates: medium promoter BBa_J23110 (844 arbitrary units [16]). Plates were incubated for 23 h at the temperatures indicated followed by 22 h at room temperature. The median intensity above background of colonies +/− the standard deviation is given above each plate half (see Additional file 1: Figure S1)

*E. coli* strain MG1655 was favored over DH5α as the host for CP quantitation studies due to the more uniform size of colonies on agar. Growth of transformed MG1655 gave color intensities of colonies or confluent streaks across the plate that were uniform and stable (i.e. the cells maintained their color). Expression from medium- versus high-copy-number plasmids was clearly distinguishable by eye (e.g. Fig. 2a). For a finer test of quantitation, we chose two of the darker CPs because the three yellow CPs (amajLime, amilGFP and fwYellow in Fig. 1a left) exhibited low color contrast compared with yellowish wild-type *E. coli* and Lysogeny Broth (LB) agar. CP colony color was compared on a high-copy-number plasmid from two promoters reported to differ in activity by only twofold (see Fig. 2b legend). Distinction by eye was aided by a slightly lower growth temperature (Fig. 2b), while software analysis of individual colonies in the photograph clearly demonstrated more intense colors from the medium (more active) promoter at both temperatures (Additional file 1: Figure S1). These results demonstrate the potential of CPs as quantitative reporters of gene expression.

### Different stabilities and growth rates of strains bearing CP plasmids

It has been noted that tetrameric FPs that aggregate substantially can be somewhat toxic to bacteria [23]. In contrast, the ATUM information sheet lists the toxicity in *E. coli* of each of their CPs encoded by a high-copy plasmid as “not observed” [15] and other CP labs have not reported cytotoxicity. Yet high expression of CPs is required for strong cell coloration (i.e. CP gel bands that are visible in total cellular proteins stained with Coomassie blue; results not shown), and we noticed that colored liquid cultures of certain CPs expressed from high-copy plasmids in *E. coli* sometimes failed to gain the same amount of color after dilution and repeated overnight liquid culturing. This indicated that certain CPs exerted a high fitness cost when highly expressed, leading to strong selection pressure for loss of expression. This instability hypothesis was confirmed by (i) observing that plating of an overnight culture of *E. coli* expressing aeBlue gave some white colonies that were bigger than the blue colonies (Additional file 1: Figure S2A), and (ii) sequencing the aeBlue coding region in both types of colonies with the finding that loss-of-expression mutations had indeed been selected for (Additional file 1: Figure S2B and C). Although loss of CP expression was negligible after re-streaking on solid media from fresh plates (Figs. 1 and 2), stability in liquid culture is important when choosing a reporter for competition studies (see below). Therefore the stabilities of all high-copy-plasmid-encoded CPs were measured using a standard liquid culture dilution assay [24]. Surprisingly, out of all 14 CPs, only fwYellow and amilGFP were very stable on high-copy plasmids in liquid culture (Additional file 1: Table S1). This is unfortunate because these two CPs were the hardest to distinguish from wild-type *E. coli*: fwYellow was not much darker and assaying amilGFP required a UV lamp. Nevertheless, the darker CPs tsPurple and meffBlue exhibited good stability through two overnight cultures (although meffBlue developed color slowly), and a large majority of the CP cultures were colored after one overnight culture. CP genes were also integrated into the *E. coli* chromosome because this generally provides higher genetic stability than carrying them on a plasmid (Additional file 1: Figure S3A). A stronger promoter was used for the integrants because there would only be one gene copy per cell. The two chromosomally-integrated CPs had less intense colors (Additional file 1: Figures S4 and S5) than the high-copy-plasmid-borne versions due to being single copy genes, but were very stable as expected (Additional file 1: Table S1).

**Fig. 3:**
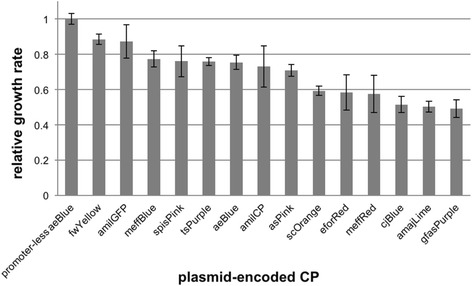
Growth rates of *E. coli* expressing each of the 14 different CPs from high-copy plasmids. The early exponential growth rates are shown as relative values compared with the control strain (promoter-less aeBlue plasmid that also lacked a ribosome binding site). Error bars are standard deviations

The relative fitness costs of the high-copy-plasmid-borne CPs in liquid culture were investigated more quantitatively by measuring early logarithmic growth rates (Fig. 3). All CPs conferred a fitness cost when over expressed, with some having effects as big as 50%. Similar-sized effects have been reported for eGFP and other proteins [25]. As expected, growth rates correlated well with the relative stabilities in Additional file 1: Table S1 (compare rates with the numbers of the most-highly-colored replicates remaining after two overnight cultures).

### CP mutagenesis creates different-colored markers having the same fitness costs in competition assays

In order to measure the fitness advantage or cost of a mutation of interest (e.g. one conferring antibiotic resistance), competition assays are performed between isogenic cell lines that differ by this mutation and as few other genetic changes as possible [26]. Such experiments require a unique marker in each cell line and have benefited from using two different CPs [6] as it allows visual assessment. In that publication, purple-blue amilCP was used in one *E. coli* cell line versus yellow amilGFP in the competing line with the assumption that both plasmid-borne genetic markers have the same fitness cost [6]. However, Additional file 1: Table S1 and Fig. 3 suggest caution when making such assumptions. We thus aimed to synthesize different-colored versions of the same CP, as they are expected to have very similar fitness costs.

**Fig. 4:**
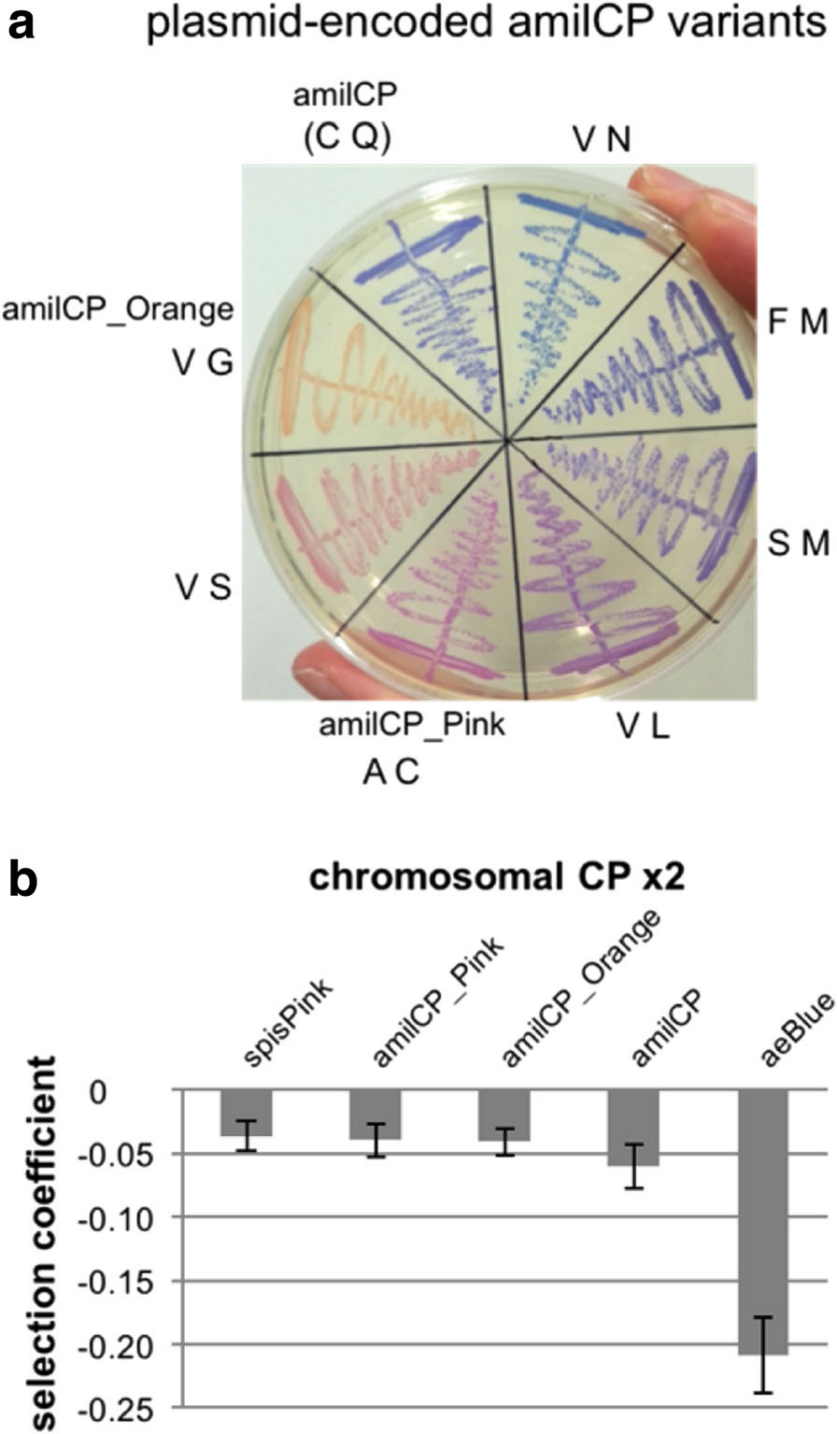
Mutagenesis of amilCP to create different-colored markers with the same fitness effects in competition assays. **a** Positions C64 and Q65 of amilCP (GFP numbering; Table 1) in pSB1K3 plasmid were mutagenized randomly and different-colored bacterial colonies (results not shown) were chosen for streaking on an LB kanamycin plate. **b** Double integrants were made of each of three amilCP variants from **a**, as well as of spisPink and aeBlue, and their fitness effects were measured by direct competition in LB medium with isogenic wild-type *E. coli*. Analysis was by plating and counting of colored versus white colonies. Selection coefficients with negative values show that the expression confers a fitness cost for the cells. Error bars are standard deviations

The absorption maxima of many FPs and CPs have been altered through random and site-directed mutagenesis, with successful hot spots being the chromophore region, residues contacting the chromophore in the tertiary structure, and even quaternary interactions between protein monomers [1, 2]. As results have been empirical rather than predictive, we focused on the chromophore region for mutagenesis. This strategy built on knowledge that:

(i) random mutagenesis of GFP’s chromophore region caused red shifts in absorbance maxima when as few as two adjacent amino acids were changed: position 65 of the chromophore (S65-Y66-G67) and position 64, with most mutants including a change at position 65 [27],
(ii) position 64 is a highly-conserved Phe in FPs whereas positions 64-65 vary considerably in CPs [28] (see alignment in Table 1), and
(iii) one of the two mutations necessary in combination to change CP color from purple to blue was at position 64 [2].
  Thus, positions 64-65 were randomly mutagenized jointly in both amilGFP and amilCP, with these CP choices based on their colors and effects on growth rate (Fig. 3; amilGFP had the equal-lowest growth effect while amilCP had the equal-lowest effect of the dark, fast-maturing CPs). Although the amilGFP mutagenesis procedure worked (as judged by sequencing), the colonies did not exhibit altered colors and almost all lost their fluorescence (results not shown). In contrast, the double mutants of dark purple-blue amilCP exhibited different-colored colonies at a surprisingly high frequency of 10% (Fig. 4a). The only color clearly not produced was green, which is also the only color missing from our CP rainbow palette (Table 1). But a green amilCP mutant (N170I in GFP numbering) has been registered recently, although color development was slow (BioBrick K1996005 [16])). While mutagenesis results for GFP homologs remain unpredictable and difficult to rationalize, our alterations of CP color complement the successes of others [2, 9, 20, 28–31]

Finally, we set up competition assays to determine if the different-colored variants of amilCP had the expected, desired, equivalent fitness costs. Given the lower stabilities of high-copy CP plasmids compared with chromosomal integrants (Additional file 1: Table S1), the latter were preferred, provided we could solve the problem of lower color intensities of the single integrants. We therefore integrated two copies of each of several CP genes, changing the codon bias of the second copy to prevent homologous recombination (Additional file 1: Figure S3B; note that codon optimizing of amilCP increased its color intensity by 50% based on quantitation of single colonies of single integrants in Additional file 1: Figure S3C). These double integrants resulted in highly-colored colonies (Additional file 1: Figure S4) and pellets (Additional file 1: Figure S5B and C). A camera module was custom-assembled to enable measurements on different days under identical conditions (Additional file 1: Figure S5A). Fitness costs of the double integrants were measured by direct competition with wild-type *E. coli* and plating (Fig. 4b). The fitness costs of the amilCP color variants were indeed highly similar, demonstrating that they are ideal markers for competition assays.

## Conclusions

Our engineering of 14 eukaryotic CP gene sequences into a palette of *E. coli* BioBricks, together with comparisons of color intensities, maturation times and fitness costs, should simplify and expand CP study and applications. While the unexpectedly large variation in fitness costs cautions the use of high-copy-plasmid-borne CPs as markers in competition assays [6], this problem was addressed by creating versions of the same CP with very different colors and integrating two gene copies into the chromosome. Like the FPs [17], there was no single plasmid-borne CP that combined all of the most desirable features, so this study should help direct future engineering efforts.

## Methods

### Plasmids

Plasmids pGEM-T-11 and pGEM-T-14 encoding native amilGFP and amilCP sequences were gifts from Jeffrey H. Miller at UCLA [2, 6]. Plasmids CPB-45-441, FPB-22-441 and CPB-38-441 encoding scOrange, fwYellow and tsPurple were purchased from DNA2.0 Inc. (now ATUM, CA, USA) [15]. Plasmid vectors pSB3K3, pSB1K3 and pSB1C3 [32] (nomenclature pSB1, high copy = 100-300 for origin pMB1; pSB3, medium copy = 20-30 for origin P15A [16]; K, kanamycin^R^; C, chloramphenicol^R^; ^R^ = resistance), constitutive promoters BBa_J23110 and BBa_J23116 (Fig. 2b) and ribosome binding site BBa_B0034 were obtained from the Registry of Standard Biological Parts [16].

### Codon optimization, gene synthesis, plasmid cloning and mutagenesis

Of the 14 CPs expressed from plasmids or first copy integrants, all but the native amilGFP and amilCP genes were codon optimized for expression in *E. coli* and synthesized commercially (Additional file 1: Figure S6). All 14 were flanked by standard BioBrick RFC10 restriction sites to facilitate cloning [33]. Sequences of meffRed, asPink, spisPink, amajLime, meffBlue and gfasPurple were codon optimized using GenScript’s OptimumGene™ algorithm and synthesized by GenScript USA Inc. (NJ, USA). Sequences of eforRed, cjBlue and aeBlue were codon optimized and synthesized by Bioneer Corporation (South Korea). Sequences of scOrange, fwYellow and tsPurple, previously codon optimized [14] and synthesized by DNA2.0 Inc., were PCR amplified from the CPB-45-441, FPB-22-441 and CPB-38-441 plasmids. The amilGFP and amilCP genes were PCR amplified from plasmids pGEM-T-11 and pGEM-T-14. Inverse PCR mutagenesis [34] was used to change the chromophores of amilGFP (primers *NNN*TATGGAAACCGTTGCTTC and *NNN*GACTGATGACAGTATGTCAAAGG) and amilCP (primers *NNNNNN*TACGGAAGCATACCATTCACC and CTGTGGTGATAAAATATCCCAAG) and to remove their illegal BioBrick restriction sites [32]. All CP DNA sequences (Additional file 1: Figure S6 and Fig. 4a) were assembled with constitutive medium promoter BBa_J23110 (unless otherwise stated) and ribosome binding site BBa_B0034 in plasmids pSB3K3, pSB1K3 and/or pSB1C3. C-terminal His_6_-tagged versions of CPs were used in Fig. 2b (to provide the ultimately unnecessary options of Western blotting for CP detection, and easy protein purification). DNA sequencing was by SciLifeLab (Uppsala).

### CP expression from plasmids

Unless otherwise stated, *E. coli* MG1655 was transformed with the high-copy plasmid pSB1C3 encoding the CP from the constitutive medium promoter BBa_J23110 and grown on LB chloramphenicol agar plates for ~ 20 h at 37 °C and in LB chloramphenicol broth for ~ 18 h at 37 °C with shaking. Visualization was done in ambient light.

### Quantitation of colony darkness

The brightness of the center of the colonies as well as the agar background were digitalized using the eyedropper tool of Adobe Photoshop CS6, with darkness calculated as 100% - brightness % (see Additional file 1: Figure S1 for details). Quantitation can also be performed with the free software combination of ImageJ and Java (used on Additional file 1: Figure S3C).

### Stability assay

Stability assays were performed with high-copy plasmids as described for “Plasmid stability, liquid experiment” [24]. In ~ 10 replicates, 1 mL of LB medium was inoculated with a single CP colony for overnight incubation under chloramphenicol (30 μg/ml) selection. Then, to allow ~ 10 generations of growth in each cycle, a 1000-fold dilution with LB chloramphenicol was performed every 24 h. Assays continued for ~ 40 generations unless color was lost earlier in all replicates (Additional file 1: Table S1). Visualization was done in ambient light except for amilGFP where a UV lamp was used.

### Growth rate assay

Growth rates were measured at 37 °C in LB medium containing chloramphenicol (30 μg/ml) as follows. Overnight cultures were diluted 1000-fold with LB chloramphenicol and 200 μl aliquots were transferred to 96-well plates (BRAND, Germany). The cultures of plasmid-encoded CPs (8 wells of each CP consisting of 4 biological replicates in duplicate) were grown with continuous shaking for 16 h and optical density (OD) measurements at 600 nm were performed every 5 mins using an Infinite M200 Pro plate reader (Tecan, Switzerland) according to the manufacturer’s instructions. The calculations of maximum growth rates were based on OD_600_ from 0.03-0.07 (between 1 and 9 h) where growth was observed to be exponential. Media blanks were added to each experiment, as well as a control strain carrying the same plasmid vector but without any CP expression (pSB1C3-aeBlue without a promoter). Relative growth rates were calculated by dividing the generation time of each strain by the generation time of the control strain.

### Chromosomal integration

For single integrants, the CP gene was inserted into the *E. coli* MG1655 chromosome by replacing insertion sequence IS150 (Additional file 1: Figure S3A) using bacteriophage Lambda (λ) Red recombineering as described [35]. The PCR reactions to generate linear DNA for recombineering, which included a chloramphenicol^R^ gene (Additional file 1: Figure S3A, top image), used Phusion DNA polymerase (ThermoFisher). These CP genes were transcribed from the strong synthetic promoter CP25 [36] and terminated by λ transcriptional terminator T1. For double integrants, the second CP copies were synthesized by Integrated DNA Technologies (USA) with altered codon bias (Additional file 1: Figure S6). These copies were integrated at the same locus as the first copies (Additional file 1: Figure S3B) as follows: a kan-sacB cassette (conferring sensitivity to sucrose and resistance to kanamycin) was first inserted and then this was replaced with the second copy of the CP gene under the strong apFAB46 promoter [37].

### Quantitation of pellet color intensity

Bacterial pellets were obtained by growing overnight cultures of strains at 37 °C in 2.5 mL LB (with 30 μg/ml chloramphenicol when growing plasmid-carrying strains), transferring the cultures to 2 ml test tubes, and pelleting the bacteria by centrifugation. Images of the pellets were acquired as 2592 × 1944 pixels 24-bit red, green and blue color model (RGB) jpeg images using a Raspberry Pi camera (element14) equipped with a macro lens at a fixed focus (Additional file 1: Figure S5A). Image analysis was made using Python 2.7 to quantitate the pellet colors. All images were cropped to only contain the image section in focus (80 × 80 pixels) and the median RGB pixel value was calculated. To compare the color intensity of each pellet with the color of the wild type MG1655, the Euclidean distance in a 3-dimensional RGB space was computed ($distance=\sqrt{{\left(R2-R1\right)}^2+{\left(G2-G1\right)}^2+{\left(B2-B1\right)}^2}$).

### Anaerobic growth for maturation measurement [38]

Overnight anaerobic cultures of mRFP1 [39] and CPs expressed constitutively from plasmids in at least 2 biological replicates were prepared as follows. Inoculated cultures of 40 mL LB containing chloramphenicol (30 μg/ml) in 100 mL laboratory glass bottles were purged with N_2_ gas for 1 min and quickly sealed with lids containing a cap liner, then parafilm was wrapped around the lids. Incubation was at 37 °C for 20 h with shaking and then the cultures were each poured into 200 mL conical flasks for aeration and continued incubation with shaking at 37 °C. Color was monitored by taking 1 mL aliquots at the indicated times, centrifuging at 13 krpm for 1 min and then analysing with the camera in Additional file 1: Figure S5A.

### Competition fitness cost assay

A standard protocol was used [26, 40]. Starter cultures of individual colonies of *E. coli* MG1655 or double integrants (at least 5 biological replicates) were grown overnight in LB without antibiotics. Then cultures were mixed 1:1 and serially passaged with a 1000-fold dilution every 24 h (1 μl culture in 1 ml LB), resulting in 10 generations of growth per passage. The ratios between the two competing strains were measured by plating and scoring for white and colored colonies. The strains were competed for 30-50 generations and selection coefficients were calculated using the regression model s = [ln(R(t)/R(0))]/[t] as previously described [26, 40], where R is the ratio of CP expressing strain to wild type and t is the number of generations.

## Additional file

Additional file 1: **Table S1.** Assay of stabilities in liquid cultures of the CP genes. **Figure S1.** Quantitation of darkness of individual CP colonies in Fig. 2b. **Figure S2.** Characterization of spontaneous mutants in the aeBlue coding region of the plasmids. **Figure S3.** Chromosomally-integrated CP genes. **Figure S4.** Comparison of color intensities of plates bearing single- and double-integrant CPs versus the respective plasmid CPs. **Figure S5.** Quantitation of color intensities of bacterial pellets. **Figure S6.** DNA sequences of CP coding regions. (DOCX 27998 kb)

## Abbreviations

CP: Chromoprotein
*E. coli*: *Escherichia coli*
FP: Fluorescent protein
FRET: Förster resonance energy transfer
GFP: Green fluorescent protein
iGEM: International genetically engineered machine
LB: Lysogeny Broth
NF: Non-fluorescent
OD: Optical density
PCR: Polymerase chain reaction
^R^: Resistance
RGB: Red, green and blue color model
S: Slower color development
TNT: Trinitrotoluene
UV: Ultra-violet light
λ: Bacteriophage Lambda
