# Supplementary material for: Engineering a palette of eukaryotic chromoproteins for bacterial synthetic biology
Source: J Biol Eng. 2018 May 10;12:8. doi: 10.1186/s13036-018-0100-0 (PMC5946454; doi:10.1186/s13036-018-0100-0)
Supplement: Supplementary file 1 — Table S1. Assay of stabilities in liquid cultures of the CP genes. Figure S1. Quantitation of darkness of individual CP colonies in Fig. 2b. Figure S2. Characterization of spontaneous mutants in the aeBlue coding region of the plasmids. Figure S3. Chromosomally-integrated CP genes. Figure S4. Comparison of color intensities of plates bearing single- and double-integrant CPs versus the respective plasmid CPs. Figure S5. Quantitation of color intensities of bacterial pellets. Figure S6. DNA sequences of CP coding regions. (DOCX 27998 kb) [file 13036_2018_100_MOESM1_ESM.docx]

SUPPORTING INFORMATION

**Engineering a palette of eukaryotic chromoproteins for bacterial synthetic biology**

Josefine Liljeruhm^1^, Saskia K. Funk^1^, Sandra Tietscher^1^, Anders D. Edlund^1,2^, Sabri Jamal^2^, Pikkei Wistrand-Yuen^2^, Karl Dyrhage^2^, Arvid Gynnå^1,2^, Katarina Ivermark^3^, Jessica Lövgren^3^, Viktor Törnblom^3^, Anders Virtanen^1^, Erik R. Lundin^2,4^, Erik Wistrand-Yuen^4^ and Anthony C. Forster^1,5^*

1. Department of Cell and Molecular Biology, Uppsala University, Uppsala, Sweden

2. iGEM Uppsala, Uppsala University, Uppsala, Sweden

3. Biology Education Centre at Uppsala University, Uppsala, Sweden

4. Department of Medical Biochemistry and Microbiology, Uppsala University, Uppsala, Sweden

5. Science for Life Laboratory, Uppsala University, Uppsala, Sweden

* To whom correspondence should be addressed: Anthony C. Forster ([a.forster@icm.uu.se](mailto:a.forster@icm.uu.se))

**Contents**

**Table S1.** Assay of stabilities in liquid cultures of the CP genes.

**Figure S1.** Quantitation of darkness of individual CP colonies in Figure 2B.

**Figure S2.** Characterization of spontaneous mutants in the aeBlue coding region of the plasmids.

**Figure S3.** Chromosomally-integrated CP genes

**Figure S4.** Comparison of color intensities of plates bearing single- and double-integrant CPs versus the respective plasmid CPs.

**Figure S5.** Quantitation of color intensities of bacterial pellets.

**Figure S6.** DNA sequences of CP coding regions.

**Table S1.** Assay of stabilities in liquid cultures of the CP genes.


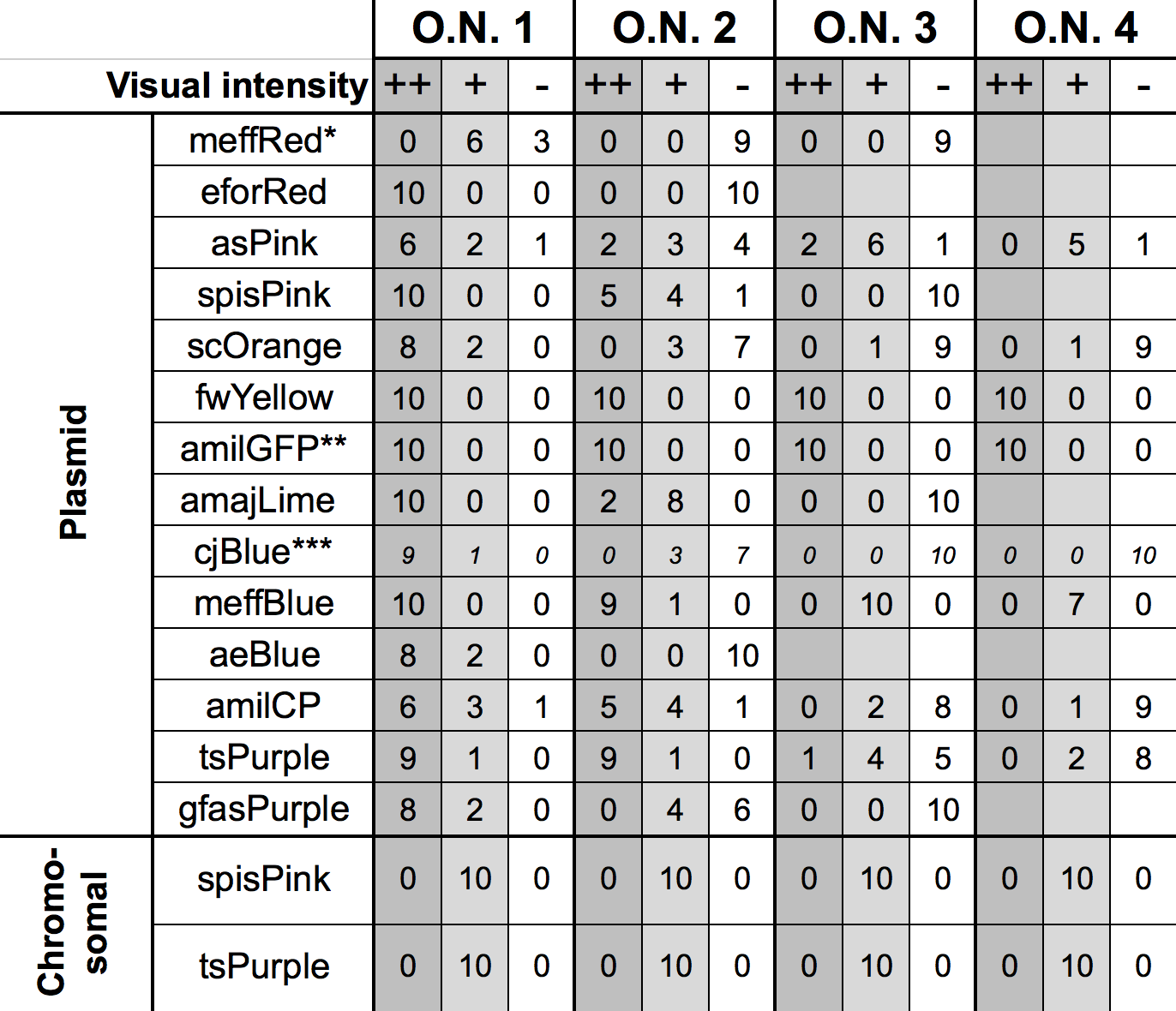


The CP genes were linked to the chloramphenicol acetyltransferase (cat) gene and expressed from a high copy plasmid (top) or as a single copy on the *E. coli* chromosome (bottom; Supporting Figure S3A). LB was inoculated by a single CP colony for overnight (O.N.) incubation 1 under chloramphenicol selection and on the next day the culture was scored visually for color intensity (~10 replicates for each overnight, with the numbers in the table below the top row corresponding to the replicates and their distribution by intensity; ++, strong color; + weak color; -, colorless). A 1000-fold dilution with LB chloramphenicol was performed for O.N. 2 and the cycle was repeated (allows ~10 generations of growth per cycle). O.N.s 3 and 4 were not performed if all cultures had lost color.

*meffRed color matured slowly, so intensity was assessed after one further night at 4°C.

**amilGFP color was difficult to differentiate from wild-type *E. coli* in LB, so expression was assessed by fluorescence on a UV lamp.

***cjBlue did not develop color in culture due to slow maturation, so fractions of O.N. cultures were plated to determine the % of blue versus white colonies. In this case, the numbers (small italics) under ++ correspond to cultures producing 90-100% blue colonies, +, 10-90% blue colonies or -, 0-10% blue colonies.

**
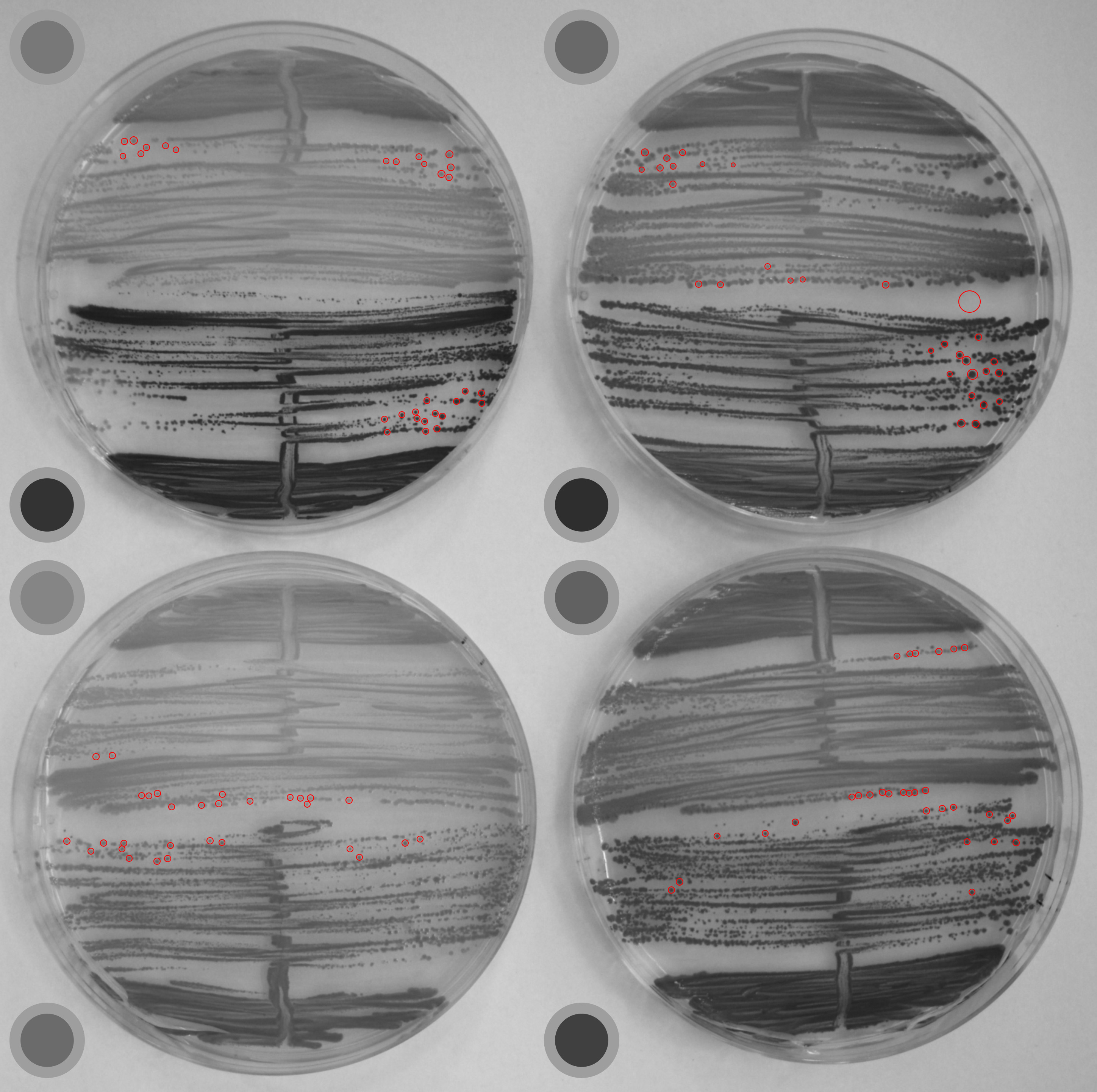
**

37 ±2

24 ±3

44 ±2

21 ±2

20 ±3

10 ±1

42 ±1

15 ±2

**Figure S1.** Quantitation of darkness of individual CP colonies in Figure 2B.

Figure 2B was converted to grey scale and representative colonies were circled in red. The darkness of the centre of each colony was calculated by the Photoshop software and the average darkness was illustrated below each streak as a circle surrounded by the measured darkness of the agar background. The difference between the centre and background yielded the number +/- standard deviation written above each streak here and in Figure 2B.

A


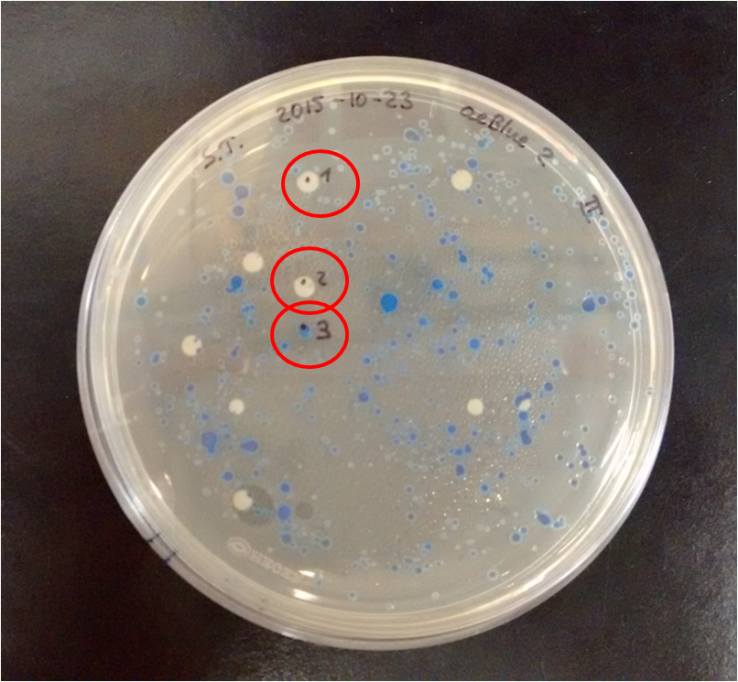


B

**Figure S2.** Characterization of spontaneous mutants in the aeBlue coding region of the plasmids.

**A.** Chloramphenicol plate of dilution of first overnight culture of *E. coli* MG1655 expressing aeBlue. Circled colonies 1-3 were analyzed by plasmid pyrosequencing.

**B.** Sequence alignment of the promoter, ribosome binding site and coding sequence from aeBlue blue colony 3 (top strands) with the sequence from mutant white colony 1 (bottom strands). A single base deletion (yellow) shifted the reading frame to a premature stop codon (*).

**C.** Alignment of aeBlue blue colony 3 sequence (top strands) with mutant white colony 2 sequence (bottom strands). The aeBlue coding region was disrupted by insertion of an *E. coli* transposon (yellow) encoding a premature stop codon (*).

**
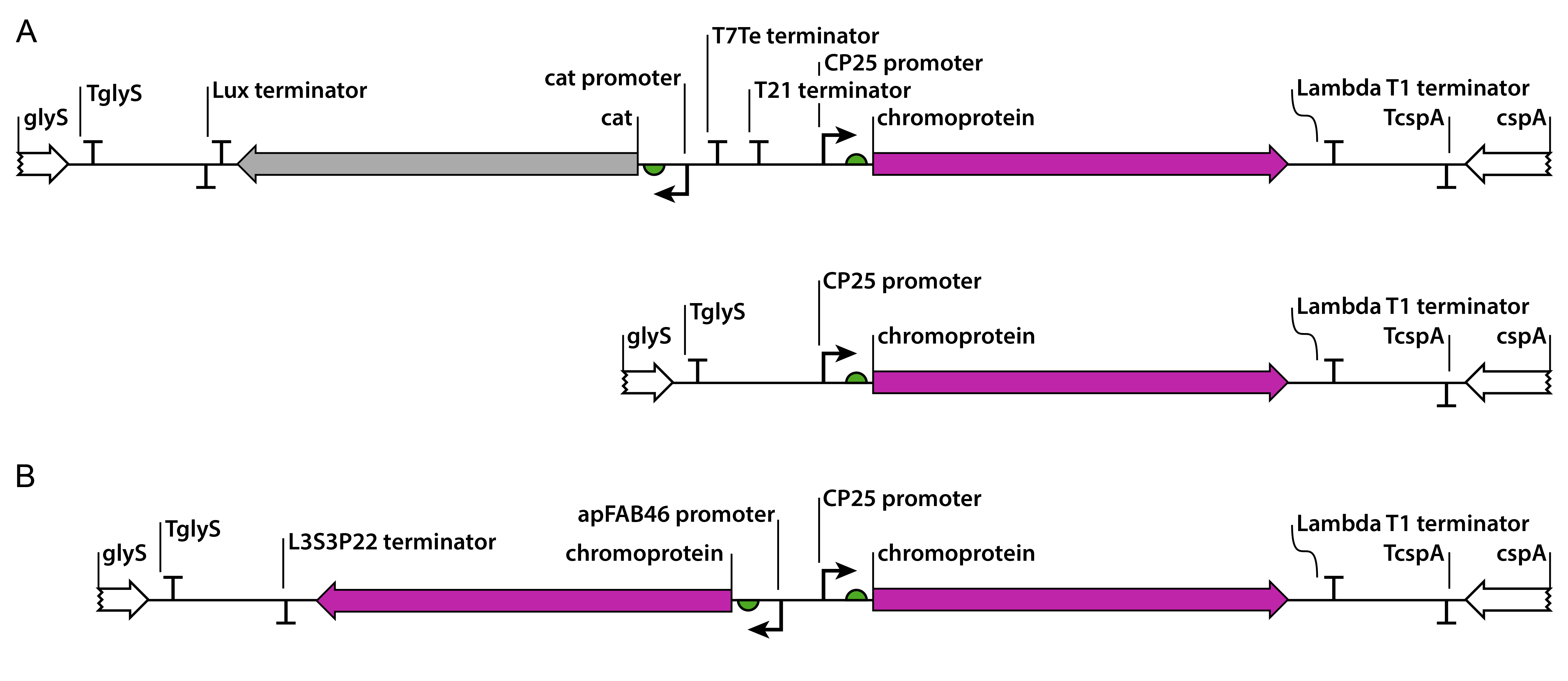
**


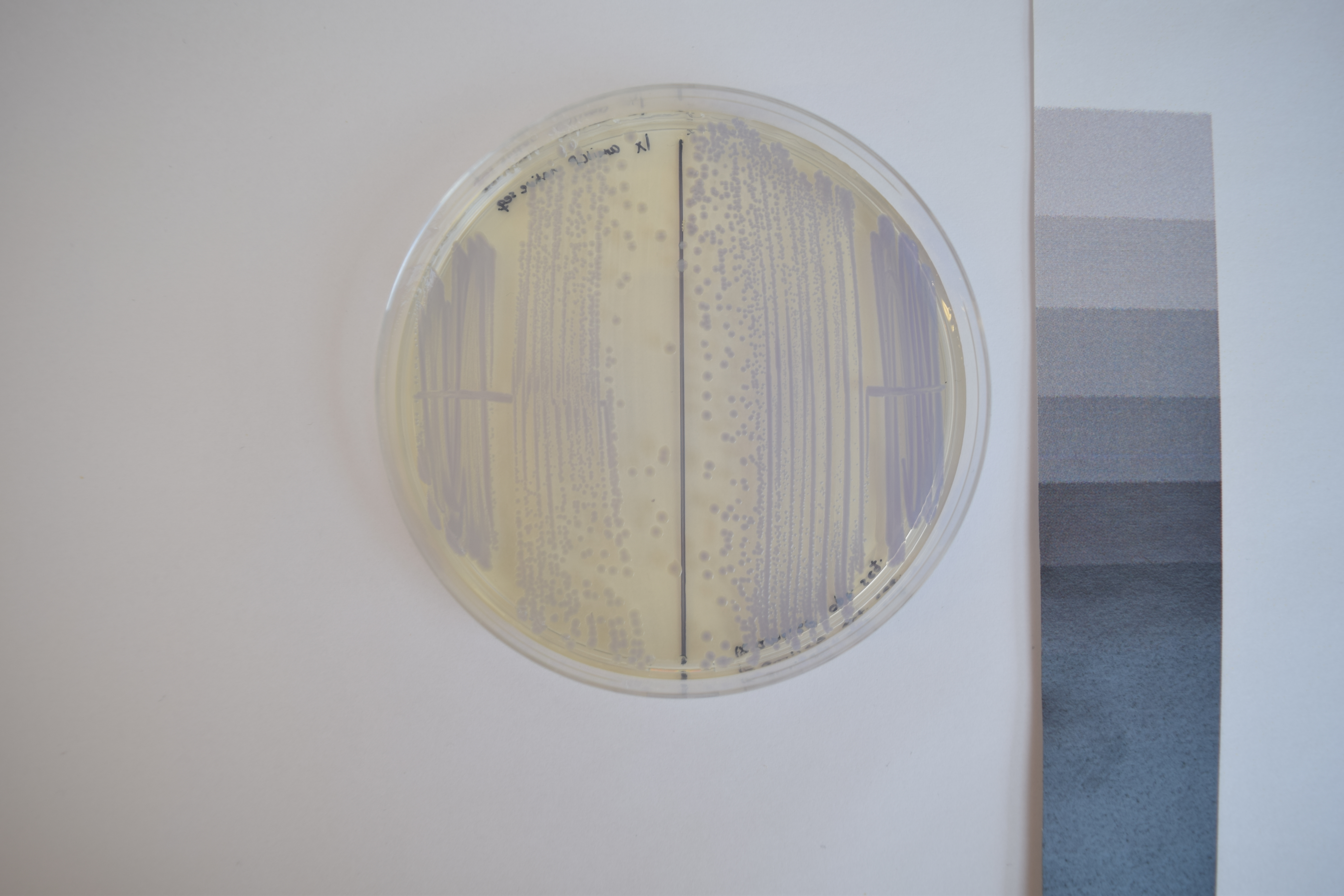


C

native codon optimized

**Figure S3.** Chromosomally-integrated CP genes.

**A.** Genetic maps of single CP integrants. Top: The chloramphenicol acetyltransferase (cat) gene (grey) together with the spisPink or tsPurple gene (purple) were inserted into the *E. coli* MG1655 chromosome to replace the insertion sequence IS150. Also shown are constitutive promoters, ribosome binding sites (green semi-circles), terminators (T) and flanking *E. coli* genes (white). Bottom: Versions with amilCP (either native sequence or codon optimized; see **C**), amilCP_Pink, amilCP_Orange or aeBlue.

**B.** Genetic map of double CP integrants constructed from strains in **A**. The two CP genes encode the same protein, but have different codon usages to avoid recombination.

**C.** Comparison of the effect of codon optimization using the single amilCP integrants shown in **A** bottom. Intensities of individual colonies on an O.N. LB plate were quantitated by ImageJ software.

**
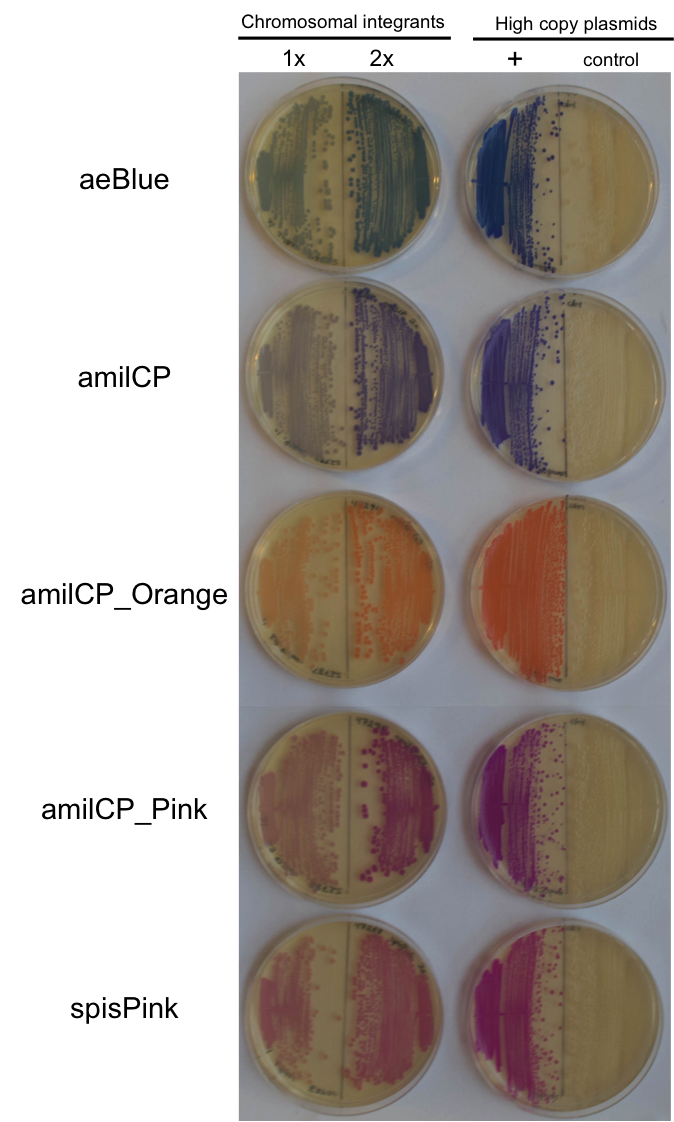
**

**Figure S4.** Comparison of color intensities of plates bearing single- and double-integrant CPs versus the respective plasmid CPs.

Plates were incubated for 23 h at 37 °C, then for 22 h at 22 °C. High-copy plasmid CPs and the control (promoter-less aeBlue plasmid that also lacked a ribosome binding site) were on chloramphenicol LB plates whereas the integrant plates lacked antibiotic.

**
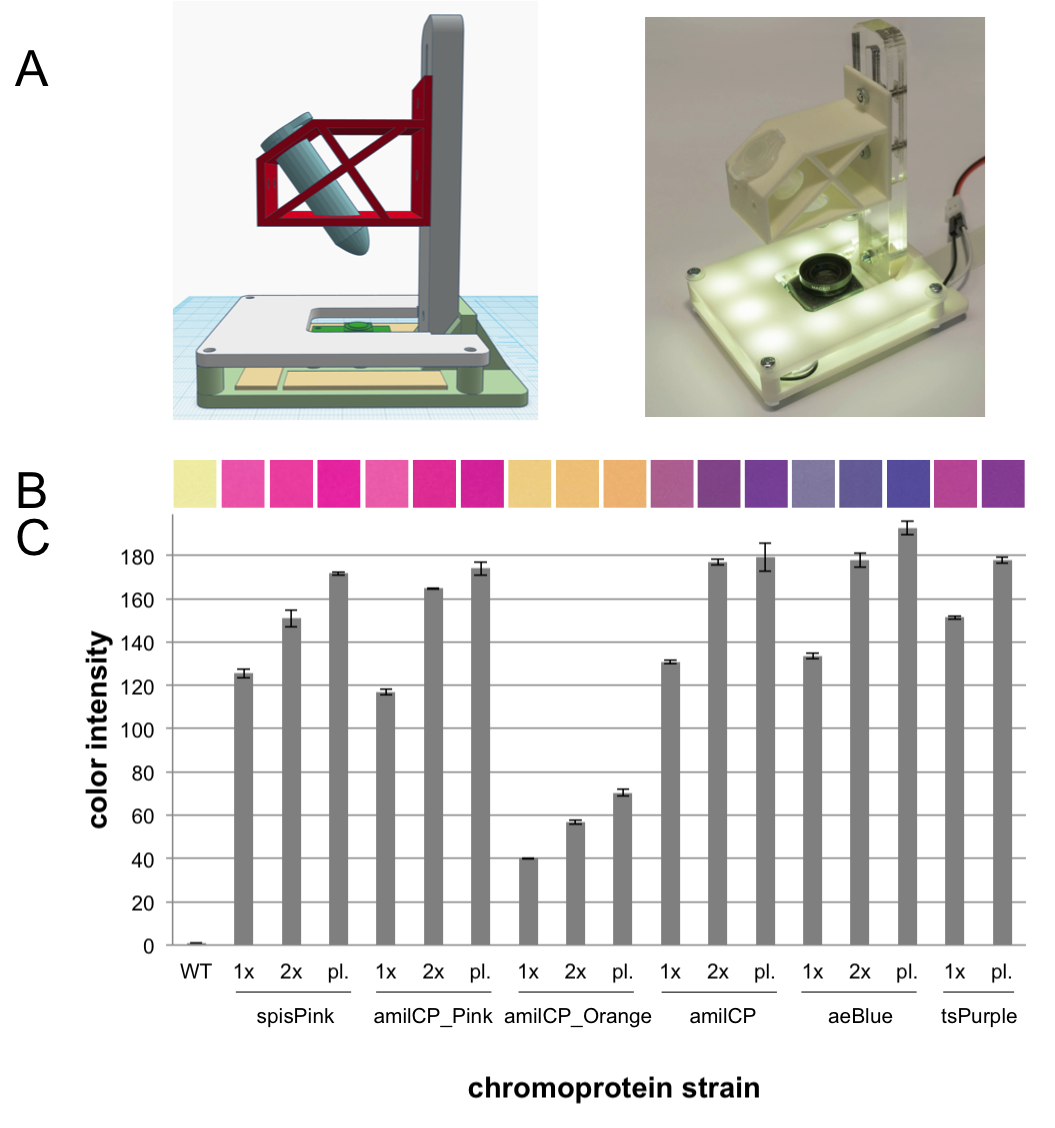
**

**Figure S5.** Quantitation of color intensities of bacterial pellets.

**A.** Drawing and photograph of a custom-built camera designed for use under a box to ensure identical parameters between different photographs of microfuge tube pellets.

**B.** Averaged colors of the centres of the pellets plotted directly below in **C**. Note that amilCP pellets look purple under these conditions.

**C.** Quantitation of pellet color intensities of different MG1655 strains expressing CPs from one integrant (1x), two integrants (2x) or a high-copy plasmid (pl.). WT = *E. coli* MG1655; its intensity was subtracted from all measurements.

**Figure S6.** DNA sequences of CP coding regions.
